# Sick-leave track record and other potential predictors of a disability pension. A population based study of 8,218 men and women followed for 16 years

**DOI:** 10.1186/1471-2458-9-104

**Published:** 2009-04-15

**Authors:** Thorne Wallman, Hans Wedel, Edward Palmer, Annika Rosengren, Saga Johansson, Henry Eriksson, Kurt Svärdsudd

**Affiliations:** 1 Uppsala University, Department of Public Health and Caring Sciences, Family Medicine and Clinical Epidemiology Section, University Hospital, Uppsala, Sweden; 2 R&D Center/Center for Clinical Research, Section of Primary Care (AmC), Sörmland County Council, Eskilstuna, Sweden; 3 Nordic School of Public Health, Gothenburg, Sweden; 4 National Social Insurance Agency, Stockholm, Sweden; 5 Uppsala University, Department of Economics, Uppsala, Sweden; 6 Department of Heart and Lung Diseases, Sahlgren Academy, Gothenburg, Sweden; 7 Department of Epidemiology, AstraZeneca R&D Mölndal, Sweden

## Abstract

**Background:**

A number of previous studies have investigated various predictors for being granted a disability pension. The aim of this study was to test the efficacy of sick-leave track record as a predictor of being granted a disability pension in a large dataset based on subjects sampled from the general population and followed for a long time.

**Methods:**

Data from five ongoing population-based Swedish studies was used, supplemented with data on all compensated sick leave periods, disability pensions granted, and vital status, obtained from official registers. The data set included 8,218 men and women followed for 16 years, generated 109,369 person years of observation and 97,160 sickness spells. Various measures of days of sick leave during follow up were used as independent variables and disability pension grant was used as outcome.

**Results:**

There was a strong relationship between individual sickness spell duration and annual cumulative days of sick leave on the one hand and being granted a disability pension on the other, among both men and women, after adjustment for the effects of marital status, education, household size, smoking habits, geographical area and calendar time period, a proxy for position in the business cycle. The interval between sickness spells showed a corresponding inverse relationship. Of all the variables studied, the number of days of sick leave per year was the most powerful predictor of a disability pension. For both men and women 245 annual sick leave days were needed to reach a 50% probability of transition to disability. The independent variables, taken together, explained 96% of the variation in disability pension grantings.

**Conclusion:**

The sick-leave track record was the most important predictor of the probability of being granted a disability pension in this study, even when the influences of other variables affecting the outcome were taken into account.

## Background

In Sweden, all residents are covered by National Social Insurance, managed by the National Social Insurance Agency, a government agency. The insurance has several components. One of these is the right to compensation for loss of income owing to sick leave from age 18 until retirement, in the majority of cases at age 65. For sick-leave periods longer than one week a sick leave certificate issued by a physician is required. The purpose of this component of the national social insurance is to provide a close to full income replacement.

Unlike the situation in most other industrialised countries, during the period studied, there was no statutory limit on how long a person could be on sick leave with compensation. As the length of the spell increases a decision will sooner or later be taken regarding rehabilitation potential. If this potential is deemed small or non-existent the sick-leave compensation is transformed to a disability pension.

From a public health point of view a disability pension as opposed to returning to work is problematic. Several studies have shown that disability pensioners have a lower quality of life [1,2], more extensive health care utilisation irrespective of underlying morbidity [3], and a poorer survival irrespective of underlying morbidity [4] than working people. Avoiding a disability pension is therefore of great public health importance.

One of the problems involved in cases of long-term sick leave is to assess the potential for return to work. Potential prognostic factors, such as age, sex, socio-economic factors, time on sick leave, and others have been investigated in several studies [5-25]. Complicating circumstances are that the utilisation of the social insurance pension is affected by political decisions, such as the compensation level, and by economic factors, such as the business cycle, with more liberal utilisation during good times than during recessions. The Swedish Council on Technology Assessment in Health Care performed an extensive literature review in 2003, showing that there were relatively few well carried out published studies on potential predictors of disability pensions [26]. Most published studies were small or focussed on particular occupational groups, special medical problems, or certain risk factors.

The hypothesis of this study was that the sick-leave track record is a strong predictor of being granted a disability pension, possibly modified by other factors. A consequence of the hypothesis would be that subjects who eventually receive a disability pension initially have short-term sickness spells not very different from those of other subjects, but their spells become successively longer, and the intervals between spells successively shorter. Ultimately, the sickness spell intervals disappear and the sick leave benefit is sooner or later converted into a disability pension.

## Methods

Data from five ongoing population studies in Sweden, with baseline investigations between 1980 and 1993, were used. Detailed information about the studies have been presented elsewhere [4]. Briefly, random samples of the specified age and sex segments of the local general populations were drawn from the national population register of a total of 10,808 subjects, of whom 8,218 (4,623 men and 3,595 women) were less than 65 years of age, still alive and had no disability pension on 1 January 1986 (baseline for this report). Of these, 6,622 subjects (3,753 men and 2,869 women) participated in the baseline examinations in the various population studies. The overall response rate was 80.6%.

Postal questionnaires were sent to some of these subpopulations, while others answered questionnaires on location in connection with a medical examination. Data from some of the questionnaires, identical in all five subpopulations, was used for this study. The information obtained included age at the baseline examination, sex, and marital status, number of persons in the household unit, education, and smoking habits. For this report, marital status was classified as married/cohabiting or not. Education was classified on a 5-point scale ranging from mandatory education only (= 1) to college or university education (= 5). Smoking habits were classified as currently smoking or not smoking. In addition, for four of the subpopulations, a 5-degree scale was available (never smoked, ex-smoker, currently smoking less than 15 cigarettes per day, 15–24 cigarettes per day or 25 cigarettes per day or more).

Information on all compensated days of sick leave for each individual in the study population from 1 January 1986 until 31 December 2002 was obtained from the National Social Insurance Agency. The data included the first and last day of each sickness spell, irrespective of type and extent of sick-leave benefit. Sick-leave duration was defined as the number of days with compensation regardless of sick-leave extent and type of benefit. Time to the next sickness spell (the interval time) was defined as the number of non-compensated days from the conclusion of one spell to the start of the next. The annual cumulative number of days with sick leave compensation was defined as cumulative number of days from 1 January to 31 December each year. The two-year cumulative number of days with sick-leave compensation was defined correspondingly, covering any two consecutive years.

Information on whether the subjects in the study population had been granted a disability pension from 1 January 1971 until 31 December 2001 was obtained from the National Social Insurance Agency. The data obtained included the decision date, diagnoses, compensation percentage (100%, 75%, 67%, 50% or 25%) and type (temporary or permanent) of disability pension. From 1 January 1986 until 31 December 2001 745 men and 753 women received a disability pension (first decision).

A detailed distribution of disability pension diagnoses in this cohort has been presented elsewhere [13]. Briefly, the four largest groups were musculoskeletal disorders (308 men, 41.3%, and 407 women, 54.1%), mental disorders (131 men, 17.6%, and 105 women, 13.9%), cardiovascular disorders (92 men, 12.4%, and 39 women, 5.2%), and neurological disorders (51 men, 6.9%, and 47 women, 6.2%). The remaining 163 (21.9%) men and 155 (20.6%) women had various other diagnoses. Twenty (2.7%) of these men and 37 (4.9%) of the women were granted a disability pension because of labour market conditions, based on legislation from 1972 introduced by the parliament to reduce unemployment in cyclical downturns, and phased out in two steps in 1991 and 1997.

Data on cause-specific mortality from January 1 of the baseline year until December 31, 2002 was obtained from the National Cause of Death Registry. The reasons for granting the disability pension and causes of death were classified according to the International Classification of Diseases (ICD), versions 8–10 [27].

Informed consent on participation in the study was obtained from all participants, oral in the early part of the study and written later on, as required first by the Research Ethics Committees at Uppsala and Gothenburg Universities and later by the National Research Ethics Board. The Committees and Board approved the study on several occasions during the data collection process.

### Statistical consideration

Data was analysed with the SAS statistical programme package [28]. In the total samples data on compensated sick leave, data on disability pensions, mortality, age and sex were 100% complete. For those who participated in the surveys data on marital status, educational level, household size and smoking habits were 98.1% complete. Summary statistics, such as means and measures of dispersion, were computed with traditional parametric methods. Simple differences between the groups regarding continuous data were tested with Student's t-test or analysis of variance, and nominal or ordinal data with the chi-square test. Since the distributions of all sick-leave data were skewed towards high values, all analyses were performed on original as well as on log-transformed data. The results were almost identical and therefore only results based on original data are presented.

The strategy of this study was to analyse the data according to a cohort design using logistic regression. However, a prerequisite for such analysis is a progressive growth of, in this case, sick-leave track data across the follow-up time. Therefore, a guiding pilot study was performed based on a nested case-referent design by which track record data growth could be assessed more easily than in a cohort design. Cases were defined as individuals who were granted a disability pension during follow up (n = 1498). For individuals who did not have a disability pension at baseline and had not received one during follow up and who survived until disability was granted for the pensioners, two sets of referents were matched to the cases, one by age, sex, and geographical area (the cities of Gothenburg, Eskilstuna, or Uppsala), and the second by age and sex only (total referent n = 2,996). The time at which the cases received their disability pension was defined as time 0 for both cases and their referents. Mean and median sick-leave duration for each month from time 0 and backwards until baseline was then calculated in the two groups. The longest follow-up periods covered 192 months. To avoid problems owing to right truncation of unfinished sick leave at time 0, and bias due to the effects of cases waiting for their disability pension decision and being on unlimited sick leave during this time, the follow-up time was ended two years before time 0.

**Figure 1:**
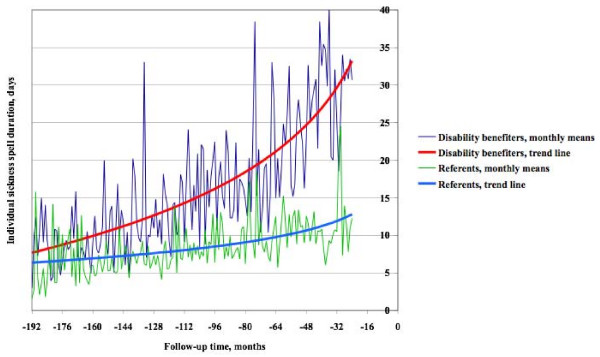
**Mean sick leave duration over time**. Mean sick leave duration among female and male cases and referents per month during follow up until two years before disability retirement (follow-up time 0) of the cases.

As shown in Figure 1, cases and referents had fairly similar mean sickness spell durations during the first few months. The differences then increased progressively during follow up, similarly for men and women, indicating that measures of sick leave-track record increased over time in both groups, but especially among those granted a disability pension, in turn indicating that logistic regression or proportional hazards regression might be appropriate analytical methods. Owing to the continuous growth of sick leave duration over time the results were independent of single long-term sick spell towards the end of the observation period.

**Figure 2:**
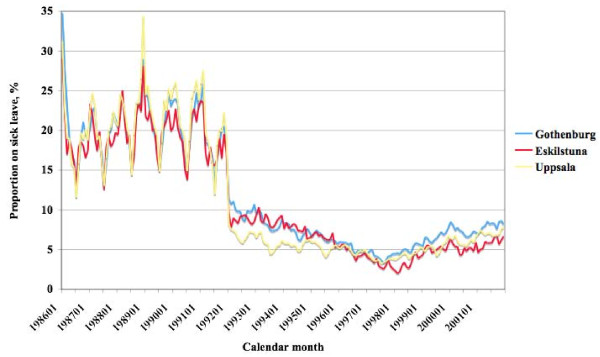
**Proportion on sick leave**. Proportion (%) on sick leave of individuals open to the possibility of sick leave certification in the cities of Gothenburg, Eskilstuna, and Uppsala per month during the 16-year study follow-up time.

In the main study design, the study population was treated as a cohort followed from 1 January 1986 until 31 December 2001. First, the monthly proportion of individuals on sick leave in relation to individuals open to the possibility of sick leave certification (still alive, age less than 65, not on a disability pension) was calculated and thus successively adjusted for non-exposure. As shown in Figure 2, there was evident variation over time, which was similar in the three geographical areas. The levels increased slowly during 1986–1992, then fell until 1998 and then increased again. During the period 1986–1992 there was strong seasonal variation, with lower levels during the summer than during the rest of the year, less pronounced from 1993 on owing to change of regulations. The variation of the proportion of subjects on sick leave across calendar time indicates the need to adjust for calendar time period as a proxy for sick leave position in the business cycle and for the effects of political decisions. In the analyses, a time variable based on calendar time period was used, coded as 1986–1992, 1993–1998, and 1998–2001.

The analyses of the influence of length of sick leave and other potential predictors on the probability of receiving a disability pension during follow up were performed using logistic regression, providing odds ratios, chi-square measures and degree of explanation based on receiver operating curves. Four analytical models were used, in which being granted a disability pension was used as outcome variable in all models. One of the exposure measures – sickness spell duration, sickness spell interval, annual sick leave days, or two-year sick leave days – was used in each model. In addition, age at baseline, marital status, educational level, household size, smoking habits, calendar time period, and geographical area were entered as explanatory variables in all models. The analyses were stratified for sex. Follow-up time was defined as number of days from baseline prior to disability pension, death, or the end of follow up, whichever came first. The latter two were thus censoring events. To account for the possibility that the results depended on a long sick leave period towards the end of follow up, the analyses were repeated with right truncation of follow-up time of one to ten years. The results were consistently the same.

The analyses were performed in two types of dataset. In the analyses using sickness spell duration and interval between sickness spells as exposure measures the observations consisted of single sickness spells updated with outcome, follow-up time and explanatory variables. In the analyses of annual sick leave days and two-year sick leave days the observations consisted of cumulative annual sick leave days or two-year sick leave days, respectively, updated with outcome, follow-up time and explanatory variables. A multilevel analysis indicated no evidence of dependence between observations within the same individual.

Only two-tailed tests were used. To account for the many analyses performed, p-values < 0.005 were regarded as statistically significant. Accordingly, 99.5% confidence intervals were used.

## Results

### Characteristics of the study population

The characteristics of the study population are presented in Table 1. The 8,218 subjects had a total of 97,160 sickness spells, and generated 109,369 person years of observation. A total of 883 subjects (420 women and 463 men) had no registered sickness spells during the study period. Mean follow-up time was 6–7 years among those who received a disability pension, and 12–14 years for the referents. The sickness spell durations and the intervals between sickness spells were markedly skewed towards high values with a mean duration of 52.6 days (median 4) for retired women and 65.6 days (median 5) for retired men. The corresponding data for referents were 14.4 days (median 3) and 14.8 days (median 4).

**Table 1:** Characteristics of the study population.

	Disability pensioners				Referents					
			
	Women		Men		Women		Men		p < for difference	
	
	Mean or %	SD	Mean or %	SD	Mean or %	SD	Mean or %	SD	Pension status	Sex
No. of subjects	753		745		2.842		3878			
Person years	5072		5201		41846		57250			
Sick leave spells	11442		9453		35553		40712			
Mean follow-up time, years	6.7	4.43	7.0	4.15	12.3	4.48	13.7	3.08	0.0001	0.0001
Sickness spell duration, days										
Mean	52.6	181.8	65.6	215.2	14.4	60.4	14.8	69.7	0.0001	0.0001
Median	4		5		3		4		0.0001	0.0001
Sickness spell interval										
Mean	111.2	235.6	128.4	277.9	198.43	421.8	221.6	451.8	0.0001	0.0001
Median	44		49		73		83		0.0001	0.0001
Cumulative one-year sick leave days										
Mean	98.3	126.2	98.4	129.7	11.3	39.3	9.8	37.9	0.0001	0.0001
Median	32		28		0		0		0.0001	0.0001
Age at baseline	47.5	9.1	47.2	8.0	41.7	13.4	39.5	11.1	0.0001	0.0001
Married, %	75.4		71.0		79.9		81.7		0.0001	n.s.
Mandatory education only, %	61.5		51.2		37.5		29.1		0.0001	0.0001.
Household size	2.7	1.2	2.5	1.2	2.9	1.3	2.9	1.3	ns	n.s.
Smokers, %	39.6		44.2		29.8		27.2		0.001	n.s.

Descriptive statistics of the study population.

Sickness spell duration and the cumulative number of sick leave days increased by age. The mean and median sickness spell durations were 16.4 and 3 days among those aged 34 or less, 24.5 and 4 days among those aged 35–44 years, 34.0 and 4 days among those age 45–54, and 33.3 and 4 days among those aged 55–64 years. The corresponding means and medians for cumulative annual sick leave days were 15.7 and 0 days, 17.5 and 0 days, 26.0 and 0 days, and 30.3 and 1 day.

The interval means showed a corresponding difference between disability pensioners-to-be and referents as the duration, but in the reverse direction. The cumulative annual sick leave days showed similar variation between pensioners and referents as the sick leave duration data, and so did cumulative two-year sick leave days (data not shown). All four sick leave measures showed significant differences between pensioners and referents, and between the two sexes.

Those who became disability pensioners were less often married, had less education, and were smokers to a larger extent than referents. There were no significant differences between the groups regarding household size.

### Effects of potential predictors on disability pension probability

After adjustment for the influence of age, men had lower odds than women for disability pension (OR 0.83, CI 0.74–0.93). After adjustment for age, marital status, education, household size, smoking habits and calendar time period there were no significant differences in disability pension rate between women and men. However, since the effects of the adjustment variables were somewhat different among women and men all further analyses were stratified by sex.

In the upper half of Table 2 the effects of individual sickness spell duration and other potential predictors on the probability of receiving a disability pension during follow up is presented. For both women and men, sickness spell duration, age and calendar time period independently increased the probability of disability pension, while high educational level decreased the probability. The probability of receiving a disability pension was lower in Eskilstuna than in Uppsala and Gothenburg. All these effects were highly significant (p < 0.0001). The strength of the predictors is reflected by the chi-square values (all with 1 degree of freedom). Among women the most powerful predictors were duration, calendar time period and age in that order, and among men duration, age and calendar time period. The model involving interval between sickness spells showed similar results as the one with sickness spell duration, except that the effect of the interval variable was the reverse of that of duration (data not shown).

**Table 2:** Effect of predictors.

	Women			Men		
	
	OR	99.5%CI	Chi-square	OR	99.5%CI	Chi-square
**Single sickness spell duration**						
Duration by 10 day-periods	1.08	1.08–1.09	1191.8	1.08	1.07–1.09	836.0
Age by 10-year age groups	3.35	2.59–4.34	174.2	3.73	2.80–4.98	165.4
Calendar time period				1		
1986–1992 vs 1999–2001	0.14	0.08–0.25	219.1	0.23	0.10–0.51	98.9
1993–1998 vs 1999–2001	1.37	0.77–2.45	83.5	1.72	0.78–3.82	52.1
Educational level	0.76	0.64–0.90	21.1	0.82	0.67–1.00	7.7
Married	1.43	0.92–2.22	5.2	0.75	0.46–1.22	2.8
Household size	1.00	0.82–1.21	0.001	0.88	0.72–1.06	3.8
Smokers	1.09	0.78–1.53	0.5	1.04	0.70–1.53	0.06
Gothenburg versus Uppsala	0.86	0.57–1.29	1.1	0.77	0.50–1.19	0.8
Eskilstuna versus Uppsala	-	-	-	0.43	0.21–0.87	8.3
**Cumulative annual sick leave days**						
Sick leave days by 10-day periods	1.16	1.14–1.17	1808.3	1.16	1.15–1.17	1473.0
Age by 10-year age groups	2.33	1.86–2.92	112.2	2.70	2.10–3.46	127.2
Calendar time period						
1986–1992 vs 1999–2001	0.52	0.32–0.86	42.4	0.92	0.47–1.78	5.6
1993–1998 vs 1999–2001	1.38	0.84–2.28	27.6	1.70	0.87–3.34	13.8
Educational level	0.84	0.72–0.97	11.6	0.87	0.73–1.02	5.8
Married	1.35	0.91–2.01	4.5	0.82	0.53–1.28	1.6
Household size	0.98	0.83–1.16	0.1	0.89	0.76–1.04	4.4
Smokers	1.19	0.88–1.61	2.5	1.17	0.82–1.65	1.5
Gothenburg versus Uppsala	0.88	0.60–1.29	0.9	0.80	0.53–1.21	0.8
Eskilstuna versus Uppsala			-	0.48	0.29–0.80	11.8

Effects of possible predictors for the probability of being granted a disability pension during the study follow-up time. In the two logistic regression models disability pensioner-to-be (yes/no) was used as dependent variable and the listed variables as independent variables.

The effects of the model involving cumulative annual sick leave days are shown in the lower part of Table 2. The results were similar to those for sickness spell duration. The probability increased with number of annual sick leave days, age, and calendar time period, while a higher educational level decreased the disability pension probability among women. The probability of receiving a disability pension in relation to annual sick leave days was similar in Gothenburg and Uppsala and lowest in Eskilstuna. Among both women and men the most powerful predictors were annual number of sick leave days, age and calendar time period. The fourth model, using two-year cumulative sick leave days, showed similar results (data not shown). Altogether, the exposure variables explained 94–97% of the disability pension variance, depending on sex and sick leave measure used.

**Figure 3:**
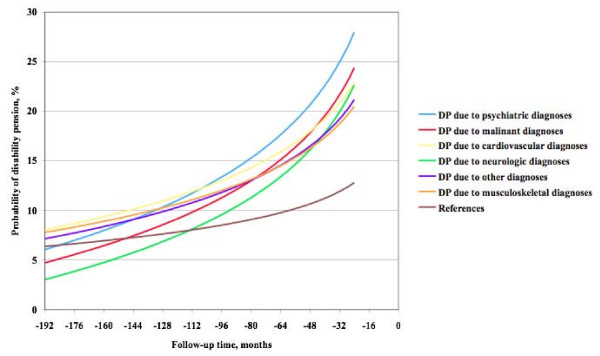
**Disability pension diagnosis and mean sick leave duration over time**. Mean sick leave duration among female and male cases and referents per month during follow up until two years before disability retirement of the cases among groups according to disability pension diagnosis.

There were no significant differences between the various disability pension diagnoses regarding the sick leave track record as tested in the cohort design. None had a significantly faster or slower progression towards disability pension, as shown in Figure 3 based on the case-referent design.

**Figure 4:**
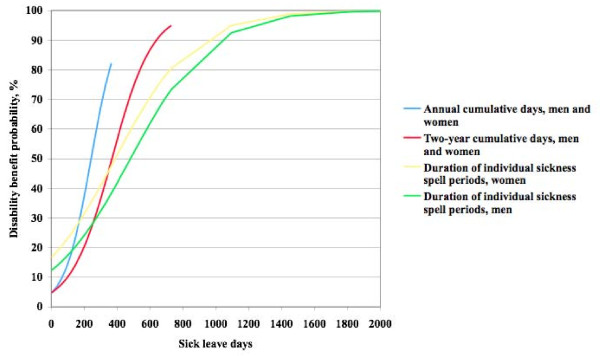
**Crude probability of disability pension**. Cumulative distribution of the proportion of men and women combined receiving disability pension by annual and two-year sick leave days, and for men and women separately by sickness spell duration during the 16-year study follow-up time with no further adjustments.

The crude influence of various levels of sick leave duration, annual sick leave days and two-year sick leave days on the cumulative disability pension distribution is presented in Figure 4. For a 50% disability pension probability a sickness spell duration of 390 days among women and 480 days among men were needed. The corresponding number of cumulative annual sick leave days was 245 days, and for cumulative two-year sick leave days 365 days, for both women and men. The corresponding number of days needed to reach an 80% probability were 730 and 830 sick leave days, 360 annual sick leave days, and 540 cumulative two-year days. Annual sick leave days thus appeared to be the most powerful predictor of receiving a disability pension.

**Figure 5:**
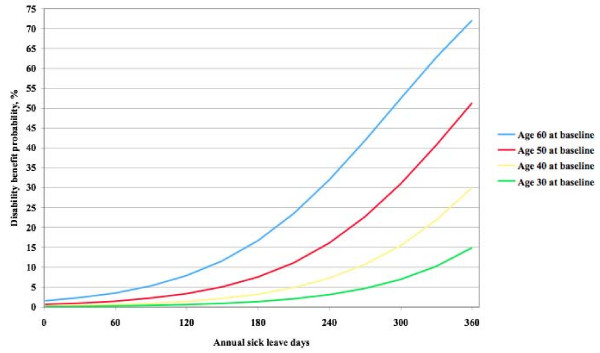
**Adjusted probability of disability pension**. Probability of being granted a disability pension in relation to number of annual sick leave days and age during the 16-year study follow-up time, adjusted for the influence of sex, marital status, education, household size, smoking habits, and business cycle.

The probability of receiving a disability pension described by annual sick leave days, age and sex, adjusted for the influence of marital status, educational level, household size, smoking habit and calendar time period is presented in Figure 5. The probability increased in terms of annual sick leave days and age, but not in terms of sex. Among the youngest subjects, the disability pension probability ranged from 0.1% with few annual sick leave days to 14.9% for individuals with the maximum number of days. The corresponding probabilities among the oldest subjects were 1.6% and 72.1%. Only the two oldest age groups reached a 50% probability of disability pension.

## Discussion

The study hypothesis was confirmed. Compared with the other significant predictors of a disability pension, i.e., age, educational level, calendar time (business cycle), and geographical area, the various measures of compensated sick leave days had the highest predictive power, and among these annual days of sick leave had the best prognostic precision. Altogether, the exposure variables entered into the model explained 94–97% of the variation in disability pensions, which is extremely high.

The study was performed in five large random samples from the general population. The participation rate was satisfactory. We had access to survey data from all the studies, and the survey data used in this study was collected using the same validated instruments in all the underlying studies. The data loss due to missing data among respondents was negligible. Sick leave, disability pensions and mortality data were all obtained from official national registers. Therefore we have no reason to believe that our results are affected by selection or other bias to such an extent that our conclusions would be affected.

Several variants of the outcome measures were tested, such as duration and annual sick leave days, accumulated durations and annual sick leave days so far (in relation to date of current sickness spell or year), and mean accumulated duration or annual sick leave days. Duration and annual sick leave days yielded the best predictions. The increase in sickness spell duration and annual sick leave data over time indicated that logistic regression and proportional hazards regression might be appropriate analytical methods. Both were used in models involving sickness spell duration, and gave similar results. In models involving annual sick leave days, hazards regression could not be used since there was no natural time variable. For this reason logistic regression was used throughout.

The advantages of the present study include a large study population derived from the general population in three areas of Sweden, access to complete sick leave, disability pension and mortality data, sufficient socio-economic data for adjustment of confounding and variance reducing variables, and a longitudinal design with long-term follow up. Most studies in this area are fairly short-term and therefore seldom cover a whole business cycle. As shown in this study such cycles may have a profound influence on the utilisation of sick leave compensation. The utilisation level in a short-term study may therefore vary with the part of the cycle studied. The scope of the effects of predictors will therefore vary with the cycle. In this study we covered about one and a half cycles, which means that our effect sizes are close to the average across a business cycle.

One of the limitations of this study is that the three geographical areas used in this study did not cover northern Sweden, which might be different from the rest of the country. However, the part of the country covered by the study has approximately 75% of the national population.

It is well known that there are large differences between countries in the regulation of sick-leave compensation and the granting of disability pension. In spite of these differences, the study of predictors of utilisation of sick leave and disability pensions is an underlying research issue for the scientific community, irrespective of nationality. From a scientific point of view Swedish social insurance has the advantage of being unlimited in terms of days and population covered during the period studied. In countries with limitations, people on sick leave have to find other means of subsistence when the statutory maximum period of compensation ends, eg, go back to work, go on to unemployment support, or social assistance. When they do, their exposure to sick leave ends, the person leaves the tracking system and may be lost to follow up. In the Swedish system a person, who has limited work capacity because of illness, can be followed through the sickness insurance system until full recovery or grant of a disability pension, i.e., there is no data loss due to right truncation of exposure and outcome data.

The employer-paid compensation for the first 14 days of sick leave from 1992 on might be a source of bias, since from that time the first day of coverage by national insurance for employees was day 15. The problem cannot be solved by disregarding the first 14 days, since the unemployed and self-employed, receive all their compensation through National Social Insurance. This means that the precision of the first 1–14 days irrespective of sickness spell duration is lower than that of 15 days or more. However, the effect of this source of bias is probably small, since the probability of being granted a disability pension is practically nil at this stage of sick leave, and the first 14 days only make up a small part of the total number of days normally needed for a disability pension.

Another source of bias might be that some individuals were granted a disability pension for labour market reasons, and consequently did not have to be on sick leave for very long. Moreover, in 1990–1991 and 1995 the regulations were changed, so that the possibilities of receiving a disability pension for labour market reasons gradually disappeared, which affects the probability of disability pension among subjects 58.5 years or older from 1991 and onwards. However, this was too small a number of subjects to cause any large bias, and most of the possible effect was taken into account by our proxy for the business cycle.

A third possible source of bias might be that the National Social Insurance Agency after shorter sick leave periods than normally required during some periods granted disability pension ("drives"). However, even these beneficiaries had fairly long sick leave records, and our observation period was long enough to even out the effects of these periods of deviation from the "norm". It is therefore unlikely that the prognosis function in this report was distorted owing to this potential source of bias.

A fourth possible bias is the fact that sick-leave track record data age, and calendar year were updated successively during the follow up, whereas other predictors were measured at baseline only. On the other hand, sex, and education would not have changed if updated, whereas marital status, smoking habits and household size might have changed. The effect of updating would have been a less dominant effect for the track record and a more powerful effect for other predictors. However, firstly, as shown by the chi-square value, the predictive power increase of updating the other predictors would have to be tremendous to even come near that of the track record data, and secondly, the purpose of introducing the other predictors was mainly to make the study population more homogeneous and avoid confounding problems.

A number of studies have assessed the effects of sick leave on the probability of being granted a disability pension. Gjesdal and Bratberg in a three-year follow-up study, found that sickness spell durations of 270 days were associated with a 40% probability of disability pension, fairly close to our 285 days for women [29]. A number of other longitudinal studies, some with very large study populations, have investigated the effects of sick-leave duration on the probability of a disability pension [4,6,7,10,12,13,30-33]. They all found strong effects of sick-leave duration, but in none of the studies was the effect quantified in terms of how many days were needed to obtain a certain disability pension probability.

Numerous studies have analysed the effects of other disability pension predictors [6-8,11,15,34-37], including a review performed by The Swedish Council on Technology Assessment in Health Care [26]. These predictors include sex, age, marital status, education, smoking habits, unemployment, full or part-time work, and other factors. Generally, the studies showed that the disability pension probability increased with female sex, age, low educational level, smoking, part-time employment, and unemployment. We found that age, calendar time period as a proxy for the position in the business cycle and education were of importance, but we found no independent significant effects of sex, marital status, smoking habits or household size. The importance of the sick-leave track record measures was far greater than that of all other factors combined.

It may appear that we found a stronger effect of track record measures and a weaker effect of other variables than in most other studies. This may be due to two circumstances. First, the long-term follow up with growing sick leave measures but constants socio-economic variables, such as educational level, to some extent favoured the sick leave measures. However, the most important explanation is probably that we used Wald's chi-square to grade the importance. Others seem to have used the odds or hazard ratios, where the size depends not only on the effect but also on the grading of the variables. If we had used the odds ratios the sick leave measures had, erroneously, been in the mid-range of the rank order of importance. Our results do not differ very much from others from a qualitative point of view, but the measurement and interpretation of predictor impact differs.

We found no differences between the various causes of disability pension regarding the effects of the measures of sick-leave days, indicating that the track record effect is approximately the same whatever the underlying disease. This issue was analysed by Gjesdal and Bratberg [6] (who used sickness absence diagnoses) and Lund et al. [30]. None of them found any effects of the underlying diagnosis. The only effect of underlying diagnosis seems to be that subjects with sick-leave compensation for psychiatric diagnoses appear to run a greater risk of being granted a disability pension [5,9,13,15,31], but there is no evidence that the sick leave-track record would be different compared to groups with other diagnoses.

The main implication of this study is that it is important to avoid granting a disability pension too early in the sick-leave history. In the Swedish setting, with 180 annual sick-leave days there is still 85% probability or more of return to work, and with 365 annual sick-leave days more than 50% return to work probability among persons 50 years or younger, and a 25% probability among those 60 years or younger.

## Conclusion

In this study the sick-leave track record was the far most important predictor of the probability of being granted a disability pension. Of the possible predictors analysed in this study, cumulative annual sick-leave days had the highest precision. Among other predictors, age, educational level, and the sick-leave spell's position in the business cycle were the strongest, but far weaker than sick-leave track record. We also found support for the underlying hypothesis that the course of sick leave prior to a disability pension begins with short-term sick-leave periods followed by spells increasing in length and with shorter and shorter intervals between them, ending finally with a disability pension.

## Competing interests

The authors declare that they have no competing interests.

## Authors' contributions

All authors designed the study and collected the data in their subpopulations or provided register data. EP, TW and KS obtained additional register data. TW, HW and KS performed the analyses and drafted the manuscript. All authors participated in the discussions of the draft outline and contributed later to revision of texts, tables and figures. All authors have seen and approved the final version of the manuscript.

## Pre-publication history

The pre-publication history for this paper can be accessed here:


